# Editorial: Microbes from marine distinctive environments

**DOI:** 10.3389/fmicb.2023.1300210

**Published:** 2023-12-06

**Authors:** Shan He, Ming Ma, Slava Epstein, Yongchao Yin

**Affiliations:** ^1^Li Dak Sum Yip Yio Chin Kenneth Li Marine Biopharmaceutical Research Center, Health Science Center, Ningbo University, Ningbo, Zhejiang, China; ^2^School of Pharmaceutical Sciences, Health Science Centre, Peking University, Beijing, China; ^3^Department of Biology, Northeastern University, Boston, MA, United States

**Keywords:** microbes, marine, diversity, natural product, deep sea, enzyme, symbiosis

Marine distinctive environments, such as the deep sea, polar seas, hydrothermal vents, the mesophotic zone, holobionts, and mangroves, are unique habitats for microorganisms, with distinctive environmental properties in terms of temperature, pressure, light, and salinity. Innumerous and distinctive microbial biodiversity in these environments awaits exploration. Major advances in manned submersibles, underwater robots (e.g., ROVs and AUVs), and SCUBA deep diving have enabled extensive observation and sample collection in these environments in recent decades. These environments are important resources for bioprospecting, including drug discovery and novel enzymes. The advent of new microbial cultivation approaches, as well as metagenomics and bioinformatics tools, provide unprecedented opportunities to study microbes from these special habitats. Thus, there are continuous research and development efforts to analyze, understand, and utilize these microbial resources. This Research Topic falls between microbiology and marine sciences. The aim was to gather recent insights into microbes from marine distinctive environments.

Symbiosis is very common in marine life. In a holobiont, the host can leverage its symbiotic microbes as producers of bioactive compounds for chemical defense and other ecological purposes. Zhao et al. investigated natural products from a marine-algal-derived endophytic fungus *Aspergillus* sp. Tennessenoid A, a novel polyketide-terpene hybrid metabolite, was discovered. The compound featured an unprecedented structure, with antifungal activities against eight plant pathogenetic fungi. Hmani et al. have cultivated epibiotic bacteria of *Ulva* from the Tunisian coast. The enzymatic and antimicrobial activities of *Ulva*-associated bacteria were evaluated. The report highlighted the enzymatic activities of seaweed epibiotic bacteria, with potential uses in the industrial sector.

The deep sea is a unique environment characterized by no sunlight penetration, high hydrostatic pressure, and low temperature. Microbes inhabiting the deep sea have evolved unique biodiversity, chemical diversity, and functional diversity. Lv et al. analyzed secondary metabolites from a deep-sea-derived fungus. Two new indole diketopiperazines, along with several known analogs with anti-microbial and anti-cancer activities, were characterized. These reports revealed the potential of discovering novel microbial natural products from marine distinctive environments for future drug development.

Marine sediments are rich in organic matter. Taurine, a naturally occurring organic sulfonate, acts as an important component of organic sulfur in marine sediments, while polyvinyl alcohol is the world's highest output water-soluble synthetic polymer, exerting an adverse effect on marine ecological environments. Tan et al. cultured a *Marmoricola* sp. actinobacterium from a deep-sea cold seep, which exhibited the prominent capability of degrading and utilizing taurine and polyvinyl alcohol for energy production.

Historic shipwrecks are anthropogenic landmarks in the marine environment, some of which have become sites of seeing, visited by SCUBA divers and submersibles. However, their influence on the local geochemistry and microbiology remains largely unexplored. Van Landuyt et al. targeted a World War II shipwreck that was hit by two aerial bombs before sinking. Chemical and microbial analyses of the samples indicated that, even after 80 years, the historic shipwreck can still significantly steer the surrounding sediment chemistry and microbial ecology.

Hydrothermal vents are unique marine environments where geothermally heated water is expelled through fissures in the Earth's crust. Their environmental conditions are extreme and significantly different from those in other marine habitats, for example, high temperature and the enrichment of organic volatiles and heavy metals. Wang L. et al. investigated the functions of microbial communities from Kueishan Island hydrothermal vents using metagenomics. This study provided insights into the microbial community in this distinctive habitant and helped understand how they survive and interact in such extreme conditions. In another case, Fonseca et al. addressed the structure, diversity, and spatial distribution patterns of the bacterial community of one of the world's largest subsurface sulfidic benthic systems. The community was dominated by sulfur-associated bacteria. A spatial pattern was unveiled along the study area, to which the family Desulfobulbaceae contributed the most to the spatial variance.

New microbial cultivation approaches enable the cultivation of previously uncultured marine microbes. Ding et al. modified iChip, an *in situ* cultivation technique, and applied the new device to access the hidden microbial diversity in marine sediments. Two novel species from the family Flavobacteriaceae were successfully cultured and systematically described. In another article, two previously uncultured novel species belonging to the genus *Kordiimonas* were cultured and reported by Ye et al.. Comparative genomic analysis indicated that the genus is a rich source of homoserine lactones, some of the most important signaling molecules in the quorum-sensing system of Gram-negative bacteria.

Marine blue holes are underwater sinkholes considered to be time capsules providing evidence of Earth's history regarding past climate change, karst processes, and life evolution. The Sansha Yongle blue hole (301.19 m depth) in the South China Sea is the world's deepest blue hole. Chen et al. unveiled a distinct separation of communities in different oxic and anoxic layers in deep gradients, and significant day-night differences were detected in the upper-layer microbial community, indicating potential vertical migration.

Coral reef ecosystems are one of the most diverse and productive habitats on Earth. Microbes in reef-overlying waters are key players in maintaining this ecosystem by regulating biogeochemical and ecological processes. Liu et al. shed light on the bacterial biodiversity of coral reefs and the importance of stochastic process in structuring bacterial communities.

Seagrass forms highly productive ecosystems in coastal environments. Pan et al. demonstrated that seagrass imposed significant ecological effects on the diversity and community organization of underground microbial eukaryotes.

Marine pathogens impose challenges to aquaculture, fishery productivity, and marine conservation. Several marine habitats, such as mangroves and wetlands, can serve as aquatic filtration systems to reduce marine pathogens. The perspective by Klohmann and Padilla-Gamiño reviewed how mangroves, shellfish beds, seagrasses, and constructed wetlands can reduce pathogen pressure in coastal ecosystems.

Dissolved oxygen is one of the key factors impacting microbial community composition in marine environments. Wang J. et al. investigated variations in the bacteria community in oxygen-depleted water, providing new insights into the potential role of denitrifying bacteria under oxygen depletion in Bohai Sea for the first time. In oceanic oxygen minimum zones (OMZs), the abundance of aerobic organisms significantly decreases, and energy shifts from higher trophic levels to microorganisms while the microbial communities become critical drivers of marine biogeochemical cycling activities. Guo et al. investigated microbial community distribution patterns across oxygen gradients, including oxygenic zones (OZs), oxygen limited zones (OLZs), and OMZs.

Planktonic microbes play vital roles in marine environments. *In situ* measurement of different size-fractionated chlorophyll *a* concentrations (Chl *a*) of phytoplankton assemblages are essential for understanding the microbial phytoplankton size structure, primary production in the ocean, and the marine biogeochemical cycle. Wei et al. showed significant differences in microbial phytoplankton size structure in different sea areas through a comparative analysis of total and size-fractionated Chl *a*.

Xie et al. focused on a ubiquitous marine fungus-like protistan group, the Labyrinthulomycetes, with a huge biomass that can exceed that of bacterioplankton in coastal oceans. The report revealed their partitioning of spatial patterns and multifaceted roles in coastal marine microbial food webs. Kim et al. investigated the physicochemical properties and bacterioplankton community of rearing water in a Biofloc technology (BFT)–based *Litopenaeus vannamei* aquaculture system. The metataxonomic results indicated a highly dynamic bacterioplankton community.

The publications in this Research Topic exhibit a broad scope, covering microbial diversity, ecology, evolution, resource mining, natural products, and enzyme discovery. To summarize, the Research Topic showcases not only the diversity of themes and contributors but also the wide scales in Marine distinctive environments, from deep sea extremes to near shore ecosystems and from hydrothermal vents to the world's deepest blue hole. Research teams from eight countries (China, United States, South Korea, Denmark, Chile, Belgium, Myanmar, and Tunisia) published 18 articles in this exciting collection of research papers and perspectives. The editorial team would like to thank all the authors and reviewers for their great contributions, helping achieve the goal of the Research Topic. The oceans link us all. We believe that this Research Topic will certainly draw more attention to the intersection of microbiology and marine sciences. With these joint efforts, we can better understand and utilize microbes from marine distinctive environments.

## Author contributions

SH: Writing – original draft, Writing – review & editing. MM: Writing – review & editing. SE: Writing – review & editing. YY: Writing – review & editing.

